# Bis(1,10-phenanthroline-κ^2^ *N*,*N*′)(sulfato-κ^2^ *O*,*O*′)nickel(II) butane-2,3-diol monosolvate

**DOI:** 10.1107/S1600536812047241

**Published:** 2012-11-24

**Authors:** Kai-Long Zhong, Chao Ni

**Affiliations:** a Department of Applied Chemistry, Nanjing College of Chemical Technology, Nanjing, 210048, People’s Republic of China

## Abstract

In the title compound, [Ni(SO_4_)(C_12_H_8_N_2_)_2_]·C_4_H_10_O_2_, the Ni^II^ ion is six-coordinated by four N atoms from two chelating 1,10-phenanthroline ligands and two O atoms from an *O*,*O*′-bidentate sulfate anion, resulting in a distorted octa­hedral geometry for the metal ion. The dihedral angle between the two chelating N_2_C_2_ groups is 83.82 (12)°. The Ni^II^ ion, the S atom and the mid-point of the central C—C bond of the butane-2,3-diol solvent mol­ecule lie on a twofold rotation axis. In the crystal, the complex mol­ecules and solvent mol­ecules are held together by pairs of symmetry-related O_diol_—H⋯O_sulfate_ hydrogen bonds involving the uncoordinating O atoms of the sulfate ions. The solvent mol­ecule is disordered over two sets of sites with site occupancies of 0.450 (9) and 0.550 (9).

## Related literature

For the ethane-1,2-diol analog of the title complex, see: Zhong *et al.* (2009 ▶). For the propane-1,3-diol analog of the title complex, see: Ni *et al.* (2010 ▶). For an isotypic structure, see: Wang & Zhong (2011 ▶).
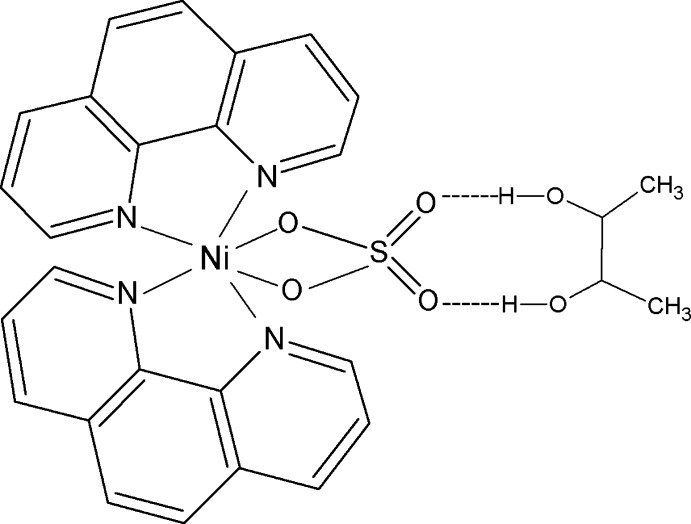


## Experimental

### 

#### Crystal data

[Ni(SO_4_)(C_12_H_8_N_2_)_2_]·C_4_H_10_O_2_
*M* *_r_* = 605.29
Monoclinic, 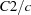

*a* = 18.147 (4) Å
*b* = 13.051 (3) Å
*c* = 13.259 (3) Å
β = 122.43 (3)°
*V* = 2650.5 (14) Å^3^
*Z* = 4
Mo *K*α radiation
μ = 0.86 mm^−1^
*T* = 223 K
0.35 × 0.30 × 0.20 mm

#### Data collection

Rigaku Mercury CCD diffractometer
Absorption correction: multi-scan (*REQAB*: Jacobson, 1998 ▶) *T*_min_ = 0.736, *T*_max_ = 1.000
6326 measured reflections
2325 independent reflections
1964 reflections with *I* > 2σ(*I*)
*R*_int_ = 0.035

#### Refinement

*R*[*F* ^2^ > 2σ(*F* ^2^)] = 0.047
*wR*(*F* ^2^) = 0.108
*S* = 1.14
2325 reflections
210 parameters
56 restraints
H-atom parameters constrained
Δρ_max_ = 0.39 e Å^−3^
Δρ_min_ = −0.39 e Å^−3^

### 

Data collection: *CrystalClear* (Rigaku, 2007 ▶); cell refinement: *CrystalClear*; data reduction: *CrystalClear*; program(s) used to solve structure: *SHELXS97* (Sheldrick, 2008 ▶); program(s) used to refine structure: *SHELXL97* (Sheldrick, 2008 ▶); molecular graphics: *XP* in *SHELXTL* (Sheldrick, 2008 ▶); software used to prepare material for publication: *SHELXTL*.

## Supplementary Material

Crystal structure: contains datablock(s) global, I. DOI: 10.1107/S1600536812047241/mw2096sup1.cif

Structure factors: contains datablock(s) I. DOI: 10.1107/S1600536812047241/mw2096Isup2.hkl

Additional supplementary materials:  crystallographic information; 3D view; checkCIF report

## Figures and Tables

**Table 1:** Hydrogen-bond geometry (Å, °)

*D*—H⋯*A*	*D*—H	H⋯*A*	*D*⋯*A*	*D*—H⋯*A*
O3—H3*A*⋯O2	0.82	1.97	2.729 (7)	154
O3′—H3′*A*⋯O2	0.82	2.03	2.769 (8)	150

**Fig. 1.**
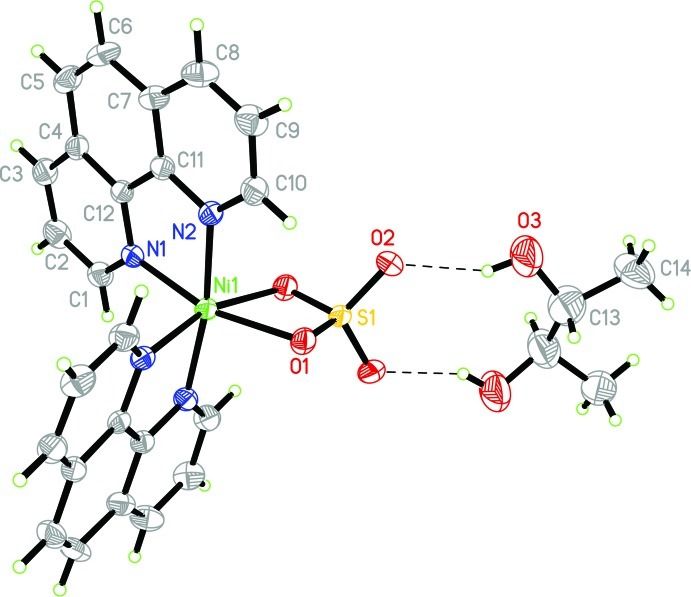
Figure 1. The molecular structure of (I) showing the atom-numbering scheme and with displacement ellipsoids drawn at the 35% probability level. The dashed lines depict O—H···O interactions. Unlabeled atoms are related to the labelled atoms by the symmetry operator (-x + 1, y, -z + 1/2).

## supplementary crystallographic information

### Comment

In the past few years, we have synthesized and reported many Ni-Phen complexes with interesting four-membered chelate rings *via* a solvothermal reaction. *e.g.* [NiSO_4_(C_12_H_8_N_2_)_2_].C_2_H_6_O_2_ (Zhong *et al.*, 2009), (II) and [NiSO_4_(C_12_H_8_N_2_)_2_].C_3_H_8_O_2_ (Ni *et al.*, 2010), (III). The title nickel complex, [NiSO_4_(C_12_H_8_N_2_)_2_].C_4_H_10_O_2_, (I), is isotypic with the previously reported cobalt(II) structure (Wang & Zhong, 2011).

The Ni^2+^ ion, the S atom and the mid-point of C—C bond of the butane-2,3-diol solvent molecule lie on a crystallographic 2-fold axis. The nickel ion is six-coordinated by four N atoms from two chelating 1,10-phenanthroline ligands and two O atoms from an O,*O*'-bidentate sulfate ion, in a distorted NiN_4_O_2_ octahedral environment (Fig. 1). The Ni—N bond distances [2.075 (3) - 2.086 (3) Å], the Ni—O bond distances [2.103 (2) Å], the N—Ni—N bite angle [79.90 (11)°], the O—Ni—O bite angle [68.06 (12)°]m and the dihedral angle between the two chelating NCCN groups [83.82 (12)°] are in good agreement with those observed in (II) and (III).

In the title compound crystal, the neutral monomeric [NiSO_4_(C_10_H_8_N_2_)_2_] complex and the butane-2,3-diol solvent molecule are connected by a pair of symmetry-related intermolecular O—H···O hydrogen bonds with the uncoordinated O atoms of the sulfate ligand (Fig.1 and Table 1).

The solvent molecule is disordered over two positions and was refined with a site occupancy ratio of 0.45:0.55.

### Experimental

The single crystals of the title compound were obtained by a procedure similar to that described preiously (Wang & Zhong, 2011), but with NiSO_4_.7H_2_O in place of CoSO_4_.7H_2_O.

### Refinement

The non-hydrogen atoms were refined anisotropically. All H atoms were positioned geometrically and allowed to ride on their attached atoms, with C—H(Ar) = 0.93 Å, C—H(CH) = 0.98 Å, C—H(CH_3_) = 0.96 Å and O—H = 0.82 Å; *U*_iso_(H) = 1.2*U*_eq_(C) and 1.5*U*_eq_(O). The solvent molecule butane-2,3-diol is disordered over two positions and was refined with 0.45 and 0.55 site occupancies.

### Crystal data

**Table:**

[Ni(SO_4_)(C_12_H_8_N_2_)_2_]·C_4_H_10_O_2_	*F*(000) = 1256
*M**_r_* = 605.29	*D*_x_ = 1.517 Mg m^−^^3^
Monoclinic, *C*2/*c*	Mo *K*α radiation, λ = 0.71073 Å
Hall symbol: -C 2yc	Cell parameters from 5284 reflections
*a* = 18.147 (4) Å	θ = 3.1–25.0°
*b* = 13.051 (3) Å	µ = 0.86 mm^−^^1^
*c* = 13.259 (3) Å	*T* = 223 K
β = 122.43 (3)°	Block, blue
*V* = 2650.5 (14) Å^3^	0.35 × 0.30 × 0.20 mm
*Z* = 4	

### Data collection

**Table:**

Rigaku Mercury CCD diffractometer	2325 independent reflections
Radiation source: fine-focus sealed tube	1964 reflections with *I* > 2σ(*I*)
Graphite Monochromator monochromator	*R*_int_ = 0.035
Detector resolution: 28.5714 pixels mm^-1^	θ_max_ = 25.0°, θ_min_ = 3.2°
ω scans	*h* = −21→19
Absorption correction: multi-scan (*REQAB*: Jacobson, 1998)	*k* = −15→14
*T*_min_ = 0.736, *T*_max_ = 1.000	*l* = −13→15
6326 measured reflections	

### Refinement

**Table:**

Refinement on *F*^2^	Primary atom site location: structure-invariant direct methods
Least-squares matrix: full	Secondary atom site location: difference Fourier map
*R*[*F*^2^ > 2σ(*F*^2^)] = 0.047	Hydrogen site location: inferred from neighbouring sites
*wR*(*F*^2^) = 0.108	H-atom parameters constrained
*S* = 1.14	*w* = 1/[σ^2^(*F*_o_^2^) + (0.0472*P*)^2^ + 2.5256*P*] where *P* = (*F*_o_^2^ + 2*F*_c_^2^)/3
2325 reflections	(Δ/σ)_max_ = 0.006
210 parameters	Δρ_max_ = 0.39 e Å^−^^3^
56 restraints	Δρ_min_ = −0.39 e Å^−^^3^

### Special details

**Table:**

Geometry. All e.s.d.'s (except the e.s.d. in the dihedral angle between two l.s. planes) are estimated using the full covariance matrix. The cell e.s.d.'s are taken into account individually in the estimation of e.s.d.'s in distances, angles and torsion angles; correlations between e.s.d.'s in cell parameters are only used when they are defined by crystal symmetry. An approximate (isotropic) treatment of cell e.s.d.'s is used for estimating e.s.d.'s involving l.s. planes.
Refinement. Refinement of *F*^2^ against ALL reflections. The weighted *R*-factor *wR* and goodness of fit *S* are based on *F*^2^, conventional *R*-factors *R* are based on *F*, with *F* set to zero for negative *F*^2^. The threshold expression of *F*^2^ > σ(*F*^2^) is used only for calculating *R*-factors(gt) *etc*. and is not relevant to the choice of reflections for refinement. *R*-factors based on *F*^2^ are statistically about twice as large as those based on *F*, and *R*- factors based on ALL data will be even larger.

### Fractional atomic coordinates and isotropic or equivalent isotropic displacement parameters (Å^2^)

**Table:**

	*x*	*y*	*z*	*U*_iso_*/*U*_eq_	Occ. (<1)
Ni1	0.5000	0.32087 (4)	0.2500	0.0327 (2)	
S1	0.5000	0.52556 (8)	0.2500	0.0338 (3)	
O1	0.56037 (14)	0.45442 (17)	0.3492 (2)	0.0396 (6)	
O2	0.45146 (16)	0.58892 (18)	0.2856 (2)	0.0478 (7)	
O3	0.4425 (6)	0.7955 (5)	0.3087 (10)	0.108 (3)	0.550 (9)
H3A	0.4578	0.7392	0.2992	0.163*	0.550 (9)
O3'	0.4035 (5)	0.7861 (6)	0.1968 (10)	0.087 (3)	0.450 (9)
H3'A	0.4351	0.7392	0.2388	0.130*	0.450 (9)
N1	0.40659 (17)	0.21783 (19)	0.1282 (2)	0.0340 (7)	
N2	0.42127 (17)	0.3083 (2)	0.3190 (2)	0.0349 (6)	
C1	0.3964 (2)	0.1793 (3)	0.0285 (3)	0.0434 (9)	
H1A	0.4366	0.1969	0.0082	0.052*	
C2	0.3278 (3)	0.1135 (3)	−0.0468 (3)	0.0505 (10)	
H2A	0.3229	0.0879	−0.1156	0.061*	
C3	0.2678 (2)	0.0866 (3)	−0.0195 (3)	0.0459 (9)	
H3B	0.2222	0.0426	−0.0691	0.055*	
C4	0.2756 (2)	0.1262 (3)	0.0841 (3)	0.0377 (8)	
C5	0.2166 (2)	0.1035 (3)	0.1210 (3)	0.0482 (9)	
H5A	0.1709	0.0580	0.0761	0.058*	
C6	0.2254 (2)	0.1464 (3)	0.2196 (3)	0.0497 (10)	
H6A	0.1860	0.1297	0.2416	0.060*	
C7	0.2946 (2)	0.2173 (3)	0.2906 (3)	0.0412 (9)	
C8	0.3046 (3)	0.2696 (3)	0.3896 (3)	0.0524 (10)	
H8A	0.2662	0.2572	0.4144	0.063*	
C9	0.3709 (3)	0.3388 (3)	0.4497 (3)	0.0512 (10)	
H9A	0.3776	0.3739	0.5152	0.061*	
C10	0.4280 (2)	0.3563 (3)	0.4125 (3)	0.0435 (9)	
H10A	0.4729	0.4033	0.4545	0.052*	
C11	0.3548 (2)	0.2399 (2)	0.2583 (3)	0.0344 (8)	
C12	0.3464 (2)	0.1929 (2)	0.1549 (3)	0.0321 (7)	
C13	0.4921 (10)	0.8683 (7)	0.3028 (11)	0.093 (4)	0.550 (9)
H13A	0.5499	0.8622	0.3764	0.112*	0.550 (9)
C13'	0.4499 (5)	0.8753 (8)	0.2301 (12)	0.077 (4)	0.450 (9)
H13C	0.4167	0.9346	0.1816	0.092*	0.450 (9)
C14	0.4532 (8)	0.9682 (7)	0.3136 (10)	0.089 (3)	0.550 (9)
H14A	0.4484	0.9651	0.3822	0.134*	0.550 (9)
H14B	0.3963	0.9781	0.2430	0.134*	0.550 (9)
H14C	0.4903	1.0244	0.3222	0.134*	0.550 (9)
C14'	0.5030 (14)	0.8961 (18)	0.3636 (13)	0.142 (7)	0.450 (9)
H14G	0.4644	0.9039	0.3918	0.213*	0.450 (9)
H14H	0.5362	0.9578	0.3791	0.213*	0.450 (9)
H14I	0.5419	0.8398	0.4043	0.213*	0.450 (9)

### Atomic displacement parameters (Å^2^)

**Table:**

	*U*^11^	*U*^22^	*U*^33^	*U*^12^	*U*^13^	*U*^23^
Ni1	0.0246 (3)	0.0300 (3)	0.0387 (4)	0.000	0.0139 (3)	0.000
S1	0.0230 (6)	0.0294 (6)	0.0429 (7)	0.000	0.0136 (5)	0.000
O1	0.0278 (12)	0.0351 (12)	0.0397 (13)	−0.0009 (10)	0.0073 (10)	−0.0017 (11)
O2	0.0398 (14)	0.0416 (14)	0.0680 (17)	0.0050 (11)	0.0329 (14)	−0.0031 (12)
O3	0.146 (6)	0.052 (4)	0.179 (8)	−0.003 (4)	0.122 (6)	−0.001 (4)
O3'	0.071 (5)	0.068 (5)	0.122 (7)	0.005 (4)	0.052 (5)	0.029 (5)
N1	0.0290 (15)	0.0309 (14)	0.0401 (16)	0.0016 (12)	0.0171 (13)	−0.0019 (13)
N2	0.0288 (14)	0.0331 (14)	0.0382 (15)	−0.0034 (12)	0.0149 (13)	−0.0050 (13)
C1	0.042 (2)	0.044 (2)	0.046 (2)	−0.0014 (17)	0.0244 (18)	−0.0082 (18)
C2	0.046 (2)	0.055 (2)	0.043 (2)	0.0032 (19)	0.0181 (18)	−0.0138 (19)
C3	0.0336 (19)	0.042 (2)	0.047 (2)	−0.0028 (16)	0.0113 (17)	−0.0106 (18)
C4	0.0285 (17)	0.0323 (18)	0.0418 (19)	−0.0010 (14)	0.0119 (15)	−0.0012 (16)
C5	0.0339 (19)	0.047 (2)	0.050 (2)	−0.0108 (17)	0.0139 (17)	−0.0034 (19)
C6	0.0329 (19)	0.060 (2)	0.055 (2)	−0.0110 (18)	0.0234 (18)	0.004 (2)
C7	0.0314 (19)	0.050 (2)	0.041 (2)	−0.0002 (16)	0.0182 (16)	0.0049 (17)
C8	0.046 (2)	0.074 (3)	0.046 (2)	−0.004 (2)	0.031 (2)	−0.001 (2)
C9	0.053 (2)	0.061 (2)	0.042 (2)	−0.001 (2)	0.0276 (19)	−0.0101 (19)
C10	0.040 (2)	0.043 (2)	0.042 (2)	−0.0033 (17)	0.0192 (17)	−0.0079 (17)
C11	0.0274 (17)	0.0331 (17)	0.0362 (18)	0.0022 (14)	0.0127 (15)	0.0017 (15)
C12	0.0252 (16)	0.0294 (16)	0.0351 (18)	0.0039 (13)	0.0117 (14)	0.0041 (14)
C13	0.118 (8)	0.075 (6)	0.114 (8)	−0.004 (6)	0.080 (7)	0.011 (6)
C13'	0.067 (7)	0.069 (7)	0.102 (8)	0.000 (5)	0.051 (6)	0.016 (6)
C14	0.107 (7)	0.090 (6)	0.110 (7)	0.020 (5)	0.084 (6)	0.018 (5)
C14'	0.153 (10)	0.180 (11)	0.130 (9)	−0.013 (8)	0.100 (8)	0.017 (8)

### Geometric parameters (Å, º)

**Table:**

Ni1—N2^i^	2.075 (3)	C4—C5	1.427 (5)
Ni1—N2	2.075 (3)	C5—C6	1.350 (5)
Ni1—N1	2.086 (3)	C5—H5A	0.9300
Ni1—N1^i^	2.086 (3)	C6—C7	1.433 (5)
Ni1—O1	2.103 (2)	C6—H6A	0.9300
Ni1—O1^i^	2.103 (2)	C7—C11	1.402 (5)
Ni1—S1	2.6714 (14)	C7—C8	1.402 (5)
S1—O2^i^	1.459 (2)	C8—C9	1.368 (5)
S1—O2	1.459 (2)	C8—H8A	0.9300
S1—O1^i^	1.499 (2)	C9—C10	1.384 (5)
S1—O1	1.499 (2)	C9—H9A	0.9300
O3—C13	1.340 (9)	C10—H10A	0.9300
O3—H3A	0.8200	C11—C12	1.435 (5)
O3'—C13'	1.362 (9)	C13—C14	1.526 (10)
O3'—H3'A	0.8200	C13—C13^i^	1.572 (14)
N1—C1	1.332 (4)	C13—H13A	0.9800
N1—C12	1.355 (4)	C13'—C14'	1.519 (11)
N2—C10	1.335 (4)	C13'—C13'^i^	1.600 (14)
N2—C11	1.363 (4)	C13'—H13C	0.9800
C1—C2	1.398 (5)	C14—H14A	0.9600
C1—H1A	0.9300	C14—H14B	0.9600
C2—C3	1.365 (5)	C14—H14C	0.9600
C2—H2A	0.9300	C14'—H14G	0.9600
C3—C4	1.401 (5)	C14'—H14H	0.9600
C3—H3B	0.9300	C14'—H14I	0.9600
C4—C12	1.412 (5)		
			
N2^i^—Ni1—N2	170.97 (15)	C3—C4—C5	124.0 (3)
N2^i^—Ni1—N1	94.23 (11)	C12—C4—C5	118.8 (3)
N2—Ni1—N1	79.90 (11)	C6—C5—C4	121.7 (3)
N2^i^—Ni1—N1^i^	79.90 (11)	C6—C5—H5A	119.1
N2—Ni1—N1^i^	94.23 (11)	C4—C5—H5A	119.1
N1—Ni1—N1^i^	99.75 (15)	C5—C6—C7	120.7 (3)
N2^i^—Ni1—O1	95.18 (10)	C5—C6—H6A	119.7
N2—Ni1—O1	92.30 (10)	C7—C6—H6A	119.7
N1—Ni1—O1	162.31 (9)	C11—C7—C8	116.9 (3)
N1^i^—Ni1—O1	96.63 (10)	C11—C7—C6	119.2 (3)
N2^i^—Ni1—O1^i^	92.30 (10)	C8—C7—C6	123.9 (3)
N2—Ni1—O1^i^	95.18 (10)	C9—C8—C7	119.9 (4)
N1—Ni1—O1^i^	96.63 (10)	C9—C8—H8A	120.1
N1^i^—Ni1—O1^i^	162.31 (9)	C7—C8—H8A	120.1
O1—Ni1—O1^i^	68.06 (12)	C8—C9—C10	119.6 (4)
N2^i^—Ni1—S1	94.52 (7)	C8—C9—H9A	120.2
N2—Ni1—S1	94.52 (7)	C10—C9—H9A	120.2
N1—Ni1—S1	130.13 (7)	N2—C10—C9	122.7 (3)
N1^i^—Ni1—S1	130.13 (7)	N2—C10—H10A	118.6
O1—Ni1—S1	34.03 (6)	C9—C10—H10A	118.6
O1^i^—Ni1—S1	34.03 (6)	N2—C11—C7	123.1 (3)
O2^i^—S1—O2	110.9 (2)	N2—C11—C12	116.8 (3)
O2^i^—S1—O1^i^	110.55 (14)	C7—C11—C12	120.1 (3)
O2—S1—O1^i^	110.56 (14)	N1—C12—C4	123.2 (3)
O2^i^—S1—O1	110.56 (14)	N1—C12—C11	117.3 (3)
O2—S1—O1	110.55 (14)	C4—C12—C11	119.4 (3)
O1^i^—S1—O1	103.47 (18)	O3—C13—C14	103.9 (8)
O2^i^—S1—Ni1	124.53 (11)	O3—C13—C13^i^	120.1 (10)
O2—S1—Ni1	124.53 (11)	C14—C13—C13^i^	113.5 (7)
O1^i^—S1—Ni1	51.74 (9)	O3—C13—H13A	106.1
O1—S1—Ni1	51.74 (9)	C14—C13—H13A	106.1
S1—O1—Ni1	94.23 (11)	C13^i^—C13—H13A	106.1
C13—O3—H3A	109.5	O3'—C13'—C14'	115.2 (14)
C13'—O3'—H3'A	109.5	O3'—C13'—C13'^i^	120.1 (5)
C1—N1—C12	117.8 (3)	C14'—C13'—C13'^i^	73.6 (11)
C1—N1—Ni1	129.3 (2)	O3'—C13'—H13C	114.0
C12—N1—Ni1	112.8 (2)	C14'—C13'—H13C	114.0
C10—N2—C11	117.8 (3)	C13'^i^—C13'—H13C	114.0
C10—N2—Ni1	129.1 (2)	C13—C14—H14A	109.5
C11—N2—Ni1	113.1 (2)	C13—C14—H14B	109.5
N1—C1—C2	122.4 (4)	H14A—C14—H14B	109.5
N1—C1—H1A	118.8	C13—C14—H14C	109.5
C2—C1—H1A	118.8	H14A—C14—H14C	109.5
C3—C2—C1	120.1 (4)	H14B—C14—H14C	109.5
C3—C2—H2A	120.0	C13'—C14'—H14G	109.5
C1—C2—H2A	120.0	C13'—C14'—H14H	109.5
C2—C3—C4	119.3 (3)	H14G—C14'—H14H	109.5
C2—C3—H3B	120.3	C13'—C14'—H14I	109.5
C4—C3—H3B	120.3	H14G—C14'—H14I	109.5
C3—C4—C12	117.2 (3)	H14H—C14'—H14I	109.5
			
N2^i^—Ni1—S1—O2^i^	−2.57 (14)	O1^i^—Ni1—N2—C10	−82.1 (3)
N2—Ni1—S1—O2^i^	177.43 (14)	S1—Ni1—N2—C10	−47.9 (3)
N1—Ni1—S1—O2^i^	−101.97 (16)	N1—Ni1—N2—C11	2.9 (2)
N1^i^—Ni1—S1—O2^i^	78.03 (16)	N1^i^—Ni1—N2—C11	−96.3 (2)
O1—Ni1—S1—O2^i^	90.01 (17)	O1—Ni1—N2—C11	166.9 (2)
O1^i^—Ni1—S1—O2^i^	−89.99 (17)	O1^i^—Ni1—N2—C11	98.7 (2)
N2^i^—Ni1—S1—O2	177.43 (14)	S1—Ni1—N2—C11	132.9 (2)
N2—Ni1—S1—O2	−2.57 (14)	C12—N1—C1—C2	−1.3 (5)
N1—Ni1—S1—O2	78.03 (16)	Ni1—N1—C1—C2	−177.4 (3)
N1^i^—Ni1—S1—O2	−101.97 (16)	N1—C1—C2—C3	0.2 (6)
O1—Ni1—S1—O2	−89.99 (17)	C1—C2—C3—C4	0.4 (5)
O1^i^—Ni1—S1—O2	90.01 (17)	C2—C3—C4—C12	0.2 (5)
N2^i^—Ni1—S1—O1^i^	87.43 (14)	C2—C3—C4—C5	179.8 (3)
N2—Ni1—S1—O1^i^	−92.57 (14)	C3—C4—C5—C6	−177.7 (4)
N1—Ni1—S1—O1^i^	−11.98 (16)	C12—C4—C5—C6	1.9 (5)
N1^i^—Ni1—S1—O1^i^	168.02 (16)	C4—C5—C6—C7	0.4 (6)
O1—Ni1—S1—O1^i^	180.0	C5—C6—C7—C11	−1.6 (6)
N2^i^—Ni1—S1—O1	−92.57 (14)	C5—C6—C7—C8	175.9 (4)
N2—Ni1—S1—O1	87.43 (14)	C11—C7—C8—C9	−0.1 (5)
N1—Ni1—S1—O1	168.02 (16)	C6—C7—C8—C9	−177.6 (4)
N1^i^—Ni1—S1—O1	−11.98 (16)	C7—C8—C9—C10	−0.3 (6)
O1^i^—Ni1—S1—O1	180.0	C11—N2—C10—C9	0.3 (5)
O2^i^—S1—O1—Ni1	−118.38 (13)	Ni1—N2—C10—C9	−178.9 (3)
O2—S1—O1—Ni1	118.39 (13)	C8—C9—C10—N2	0.2 (6)
O1^i^—S1—O1—Ni1	0.0	C10—N2—C11—C7	−0.8 (5)
N2^i^—Ni1—O1—S1	90.40 (12)	Ni1—N2—C11—C7	178.5 (3)
N2—Ni1—O1—S1	−94.66 (12)	C10—N2—C11—C12	177.2 (3)
N1—Ni1—O1—S1	−31.5 (4)	Ni1—N2—C11—C12	−3.5 (3)
N1^i^—Ni1—O1—S1	170.81 (12)	C8—C7—C11—N2	0.7 (5)
O1^i^—Ni1—O1—S1	0.0	C6—C7—C11—N2	178.3 (3)
N2^i^—Ni1—N1—C1	−12.5 (3)	C8—C7—C11—C12	−177.3 (3)
N2—Ni1—N1—C1	174.4 (3)	C6—C7—C11—C12	0.4 (5)
N1^i^—Ni1—N1—C1	−93.0 (3)	C1—N1—C12—C4	1.9 (4)
O1—Ni1—N1—C1	109.5 (4)	Ni1—N1—C12—C4	178.6 (2)
O1^i^—Ni1—N1—C1	80.3 (3)	C1—N1—C12—C11	−176.1 (3)
S1—Ni1—N1—C1	87.0 (3)	Ni1—N1—C12—C11	0.6 (3)
N2^i^—Ni1—N1—C12	171.2 (2)	C3—C4—C12—N1	−1.3 (5)
N2—Ni1—N1—C12	−1.9 (2)	C5—C4—C12—N1	179.0 (3)
N1^i^—Ni1—N1—C12	90.7 (2)	C3—C4—C12—C11	176.6 (3)
O1—Ni1—N1—C12	−66.8 (4)	C5—C4—C12—C11	−3.1 (5)
O1^i^—Ni1—N1—C12	−96.0 (2)	N2—C11—C12—N1	1.9 (4)
S1—Ni1—N1—C12	−89.3 (2)	C7—C11—C12—N1	180.0 (3)
N1—Ni1—N2—C10	−177.9 (3)	N2—C11—C12—C4	−176.1 (3)
N1^i^—Ni1—N2—C10	82.9 (3)	C7—C11—C12—C4	1.9 (5)
O1—Ni1—N2—C10	−13.9 (3)		

Symmetry code: (i) −*x*+1, *y*, −*z*+1/2.

### Hydrogen-bond geometry (Å, º)

**Table:**

*D*—H···*A*	*D*—H	H···*A*	*D*···*A*	*D*—H···*A*
O3—H3*A*···O2	0.82	1.97	2.729 (7)	154
O3′—H3′*A*···O2	0.82	2.03	2.769 (8)	150
